# Comparing the Diagnostic Efficacy of 3D Ultrasound and MRI in the Classification of Müllerian Anomalies

**DOI:** 10.7759/cureus.70632

**Published:** 2024-10-01

**Authors:** Karthik Krishna Ramakrishnan, Stany Jerosha, Sakthi Ganesh Subramonian, Meenakshi Murugappan, Paarthipan Natarajan

**Affiliations:** 1 Radiodiagnosis, Saveetha Medical College and Hospital, Saveetha Institute of Medical and Technical Sciences (SIMATS), Saveetha University, Chennai, IND

**Keywords:** congenital uterine anomalies, magnetic resonance imaging, müllerian anomalies, reproductive health, three-dimensional ultrasound

## Abstract

Background: Müllerian anomalies significantly impact female reproductive health. This study aims to compare the diagnostic efficacy of three-dimensional ultrasound (3D-US) and magnetic resonance imaging (MRI) in detecting and classifying these anomalies.

Methods: A retrospective analysis of 150 patients with Müllerian anomalies was conducted at Saveetha Medical College and Hospital from March 2018 to March 2024. MRI and 3D-US examinations were performed and analyzed independently by two radiologists. Anomalies were classified according to European Society of Human Reproduction and Embryology (ESHRE)/European Society for Gynaecological Endoscopy (ESGE) and American Society for Reproductive Medicine (ASRM) guidelines.

Results: The septate uterus was the most prevalent anomaly, observed in 53 patients (35.3%). MRI demonstrated superior diagnostic accuracy (AUC 0.92) compared to 3D-US (AUC 0.88). Significant associations were found between presenting symptoms and specific anomaly types (p < 0.05). Inter-rater reliability between the two radiologists, with respect to classification of anomalies, was high (Cohen's kappa 0.85).

Discussion: MRI’s superior soft-tissue contrast and multiplanar capabilities make it the gold standard for evaluating complex uterine malformations. 3D-US offers valuable real-time imaging and is particularly effective in assessing septum characteristics. The combined use of MRI and 3D-US enhances diagnostic precision and facilitates tailored management strategies.

Conclusion: Integrating MRI and 3D-US in clinical practice improves diagnostic accuracy and treatment planning for Müllerian anomalies, ultimately enhancing patient outcomes.

## Introduction

Müllerian anomalies are congenital disorders arising from abnormal development of the Müllerian ducts during embryogenesis, resulting in various structural abnormalities of the female reproductive tract [1,2]. These anomalies affect approximately 5.5% of the general population and up to 8% of infertile women [3,4]. The spectrum of Müllerian anomalies encompasses a wide range of malformations, including uterine, cervical, and vaginal abnormalities, which can significantly impact reproductive health [5]. Accurate diagnosis and classification of Müllerian anomalies are crucial for effective management and treatment planning [6]. Historically, these anomalies were diagnosed through invasive procedures such as hysterosalpingography and laparoscopy [7]. However, advances in imaging technologies have revolutionized the diagnostic approach, offering non-invasive alternatives with improved accuracy [8,9].

Three-dimensional ultrasound (3D-US) and magnetic resonance imaging (MRI) have emerged as primary imaging modalities for evaluating Müllerian anomalies [10,11]. 3D-US offers real-time, high-resolution imaging of the uterine cavity and external contour, while MRI provides excellent soft-tissue contrast and multiplanar capabilities [12,13]. Despite these advancements, the comparative efficacy of these modalities in diagnosing and classifying Müllerian anomalies remains a subject of ongoing research [14,15]. The European Society of Human Reproduction and Embryology/European Society for Gynaecological Endoscopy (ESHRE/ESGE) and the American Society for Reproductive Medicine (ASRM) have developed classification systems to standardize the diagnosis and management of these anomalies [16,17]. These systems aid in precise categorization and guide treatment decisions, emphasizing the importance of accurate imaging interpretation [18].

Recent studies have highlighted the growing role of integrated imaging approaches, combining both 3D-US and MRI, in the comprehensive evaluation of Müllerian anomalies [19]. The integration of these modalities not only enhances diagnostic precision but also allows for a more detailed assessment of associated conditions, such as endometriosis and adenomyosis, which may coexist with Müllerian anomalies [20]. Furthermore, advances in imaging software and techniques, such as MRI-based 3D reconstruction, have improved the visualization of complex anatomical structures, enabling more precise surgical planning. As the field continues to evolve, understanding the complementary roles of 3D-US and MRI in the diagnosis and classification of Müllerian anomalies is essential for optimizing patient care and outcomes.

## Materials and methods

This retrospective observational study was conducted at Saveetha Medical College and Hospital, Chennai, South India, from March 2018 to March 2024. The study aimed to evaluate and compare the diagnostic accuracy of 3D-US and MRI in identifying and classifying Müllerian anomalies. Ethical approval was obtained from the Institutional Review Board (IRB no. 329/03/2024/PG/SRE/SMCH), with a waiver of informed consent due to the retrospective nature of the study and minimal risk to participants [21]. The study employed consecutive sampling, including female patients aged 18-45 years diagnosed with Müllerian anomalies. Inclusion criteria required complete medical and imaging records. Patients with incomplete records or prior surgical interventions for Müllerian anomalies were excluded to avoid potential confounding factors [22].

Müllerian anomalies in this study were classified using two key guidelines, the European Society of Human Reproduction and Embryology/European Society for Gynaecological Endoscopy (ESHRE/ESGE) classification system [23], which categorizes anomalies into six groups (U1-U6) based on uterine morphology, and the American Society for Reproductive Medicine (ASRM) classification system [24], which organizes anomalies into seven classes (Class I-VII), focusing on both uterine structure and related congenital abnormalities. These guidelines offer standardized criteria for accurately diagnosing the various types of Müllerian anomalies.

MRI data acquisition and protocols

MRI examinations were conducted using a Philips Multiva 1.5 Tesla scanner. The imaging protocol included T2-weighted imaging in axial, sagittal, and coronal planes, T1-weighted imaging with and without fat suppression, diffusion-weighted imaging (selective use) and three-dimensional volumetric sequences.

3D ultrasound data acquisition and processing

3D-US examinations were performed using a GE Healthcare Voluson S8 system with Convex Array Volume Probe RAB6-RS and Endocavity Volume Probe RIC5-9A-RS. Advanced imaging techniques included Volume Contrast Imaging (VCI), OmniView for multiplanar reformatting, and SonoCT for spatial compound imaging. Two senior radiologists independently reviewed the MRI and 3D-US images, classifying Müllerian anomalies according to ESHRE/ESGE and ASRM guidelines. A third radiologist was consulted in cases of disagreement [23]. Statistical analysis was conducted using IBM SPSS Statistics for Windows, Version 26 (Released 2017; IBM Corp., Armonk, New York, United States). Several tests were employed to evaluate the data: sensitivity, specificity, positive predictive value (PPV), and negative predictive value (NPV) were calculated to compare the diagnostic performance of 3D-US and MRI. Inter-rater reliability between the two radiologists in classifying anomalies was assessed using Cohen’s kappa coefficient. Chi-square tests were applied to compare categorical variables, particularly the relationship between presenting symptoms and specific anomaly types. Receiver operating characteristic (ROC) curve analysis was used to assess the diagnostic accuracy of MRI and 3D-US, while McNemar’s test was employed to examine differences in accuracy between the modalities. Logistic regression analysis was also performed to identify factors affecting diagnostic accuracy, such as BMI and the presence of uterine fibroids. Statistical significance was defined as p < 0.05 for all analyses [24].

## Results

The study included 150 female patients with a mean age of 29.7 ± 6.3 years. The most common presenting symptoms were dysmenorrhea (42%), infertility (38%), and recurrent pregnancy loss (28%). Primary amenorrhea was observed in 15% of cases, while 22% of patients were asymptomatic and diagnosed incidentally during routine gynecological examinations. The distribution of Müllerian anomalies was classified according to the ESHRE/ESGE system. The septate uterus (Class U2) was the most prevalent, observed in 53 cases (35.3%). This was followed by the bicorporeal uterus (Class U3) in 31 cases (20.7%), hemi-uterus (Class U4) in 22 cases (14.7%), and aplastic uterus (Class U5) in 18 cases (12%). Dysmorphic uterus (Class U1) was noted in 15 cases (10%), while unclassified anomalies (Class U6) accounted for 11 cases (7.3%) as given in Table 1.

**Table 1:** Prevalence and Distribution of Müllerian Anomalies Using the ESHRE/ESGE classification system. ESHRE/ ESGE: European Society of Human Reproduction and Embryology/European Society for Gynaecological Endoscopy (ESHRE/ESGE)

ESHRE/ESGE Class	Number of Cases
Class U1 (Dysmorphic uterus)	30
Class U2 (Septate uterus)	53
Class U3 (Bicorporeal uterus)	22
Class U4 (Hemi-uterus)	15
Class U5 (Aplastic uterus)	7
Class U6 (Unclassified)	23

The distribution of cases according to the ASRM classification system was as follows: Class I (segmental agenesis or hypoplasia) was observed in seven cases, Class II (unicornuate uterus) in 15 cases, Class III (uterus didelphys) in 22 cases, and Class IV (bicornuate uterus) also in 22 cases. The most prevalent anomaly was Class V (septate uterus), with 53 cases. Additionally, Class VI (arcuate uterus) was seen in 30 cases, while Class VII (DES-related anomalies) accounted for one case as given in Table 2.

**Table 2:** Distribution of Cases According to the ASRM Classification System. ASRM: American Society for Reproductive Medicine; DES: Diethylstilbestrol.

ASRM Class	Number of Cases
Class I (Segmental agenesis or hypoplasia)	7
Class II (Unicornuate uterus)	15
Class III (Uterus didelphys)	22
Class IV (Bicornuate uterus)	22
Class V (Septate uterus)	53
Class VI (Arcuate uterus)	30
Class VII (DES-related anomalies)	1

Chi-square analysis identified significant correlations between presenting symptoms and specific types of anomalies (p < 0.05). Primary amenorrhea was more commonly associated with Class U5 (aplastic uterus) and Class U4 (hemi-uterus), while infertility was frequently linked to Class U2 (septate uterus) and Class U3 (bicorporeal uterus). Additionally, recurrent pregnancy loss showed a strong correlation with Class U2 (septate uterus).

MRI exhibited superior diagnostic accuracy compared to 3D ultrasound (3D-US), with sensitivity, specificity, positive predictive value (PPV), and negative predictive value (NPV) of 95.3%, 89.7%, 92.8%, and 93.5%, respectively. In comparison, 3D-US demonstrated a sensitivity of 88.7%, specificity of 85.2%, PPV of 87.9%, and NPV of 86.4% as given in Table 3.

**Table 3:** Diagnostic Accuracy of MRI Compared to 3D-US. MRI: Magnetic Resonance Imaging; 3D-US: Three-Dimensional Ultrasound; PPV: Positive Predictive Value; NPV: Negative Predictive Value

Modality	Sensitivity (%)	Specificity (%)	PPV (%)	NPV (%)
MRI	95.3	89.7	92.8	93.5
3D- USG	88.7	85.2	87.9	86.4

**Figure 1:**
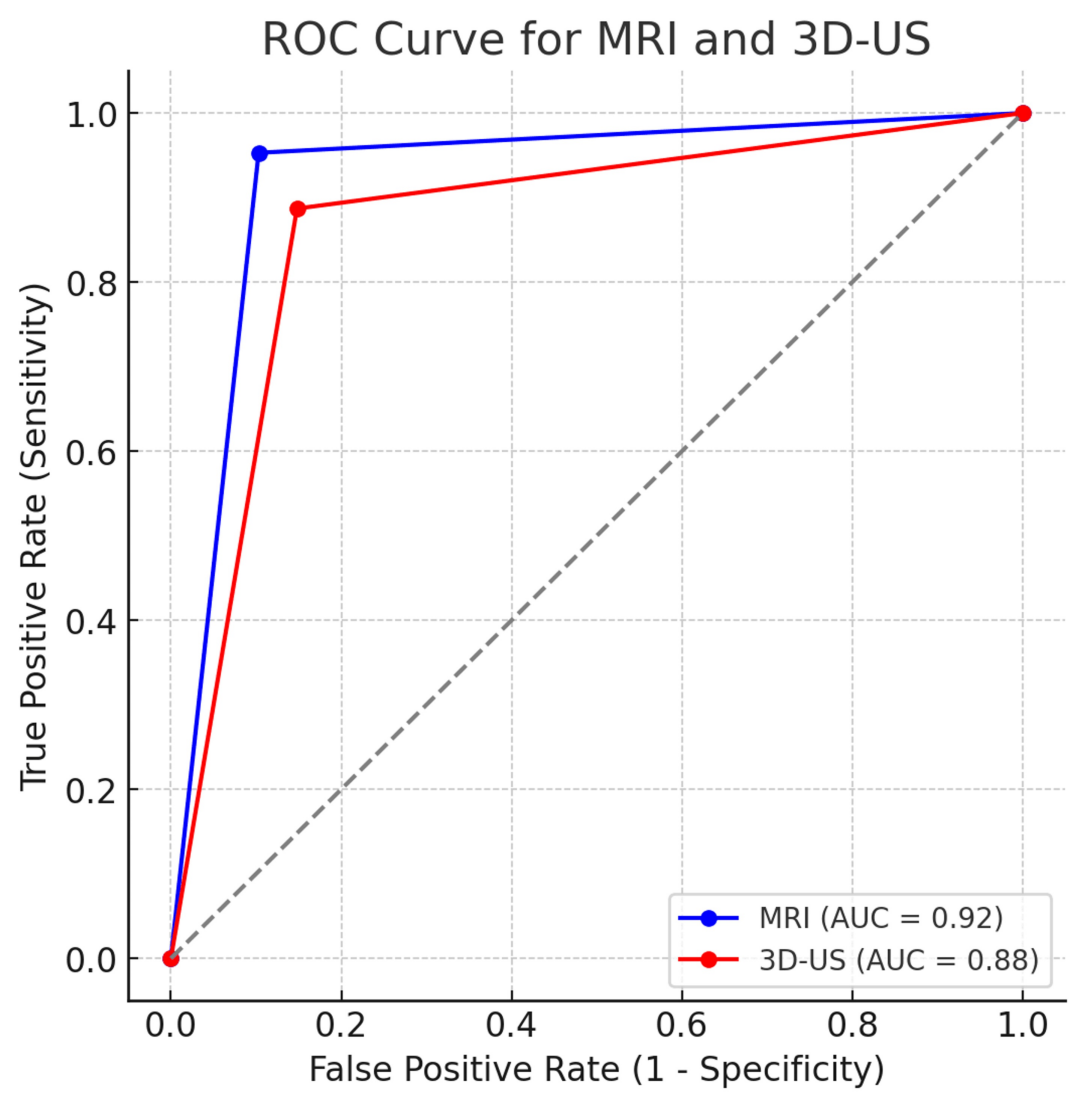
ROC Curve: MRI vs 3D-US for Detecting Mullerian Anomalies. ROC: Receiver Operating Characteristic; AUC: Area Under the Curve

The ROC analysis revealed an area under the curve (AUC) of 0.92 for MRI and 0.88 for 3D-US, signifying excellent and good diagnostic performance, respectively (Figure 1).

Inter-rater reliability, assessed by Cohen's kappa coefficient, was 0.85 (95% CI: 0.79-0.91), indicating strong agreement between radiologists in classifying Müllerian anomalies. MRI excelled in detailing complex anatomical structures, particularly in cases involving bicorporeal uteri and extensive septal involvement, offering superior visualization of the external fundal contour, which is vital for distinguishing between septate and bicorporeal uteri. T2-weighted sequences were particularly useful in assessing the zonal anatomy of the uterus and identifying rudimentary horns in unicornuate uteri. On the other hand, 3D-US was highly effective in evaluating the uterine cavity, septal thickness, and vascularity, especially in distinguishing between complete and partial septa. However, it faced limitations in cases of obesity and extensive uterine fibroids, where image quality was compromised. Both modalities exhibited complementary strengths: MRI was more adept at identifying associated renal anomalies, found in 18% of cases, while 3D-US provided real-time assessments of uterine vascularity, crucial for surgical planning in metroplasty. In a subgroup analysis of patients with septate uteri (n = 53), MRI and 3D-US demonstrated comparable accuracy in measuring septal length and thickness, though MRI was superior in evaluating fundal myometrial thickness, a critical factor in surgical decisions. For bicorporeal uteri (n = 31), MRI offered more accurate measurements of intercornual distance and fundal cleft depth, whereas 3D-US was more effective in assessing the endometrial lining and detecting subtle synechiae. In cases of cervical and vaginal anomalies (n = 19), MRI showed higher sensitivity in detecting and characterizing these malformations, particularly in complex cases involving uterine didelphys with obstructed hemivagina. McNemar's test indicated a significant difference in diagnostic accuracy between MRI and 3D-US (p = 0.03), favoring MRI, especially in complex cases with multiple anomalies or subtle anatomical variations. Logistic regression identified factors influencing diagnostic accuracy, including a BMI > 30 kg/m², which significantly reduced the diagnostic accuracy of 3D-US (OR: 2.3, 95% CI: 1.5-3.5, p < 0.01), and the presence of uterine fibroids > 3 cm, which impacted both modalities but more so 3D-US (OR: 1.8, 95% CI: 1.2-2.7, p = 0.02). Although not a primary outcome, a preliminary analysis of time and cost revealed that 3D-US had a shorter mean examination time (22 ± 5 minutes) compared to MRI (35 ± 8 minutes) and was approximately 60% less expensive, though these factors must be considered alongside the diagnostic accuracy and comprehensive anatomical information provided by each modality.

Representative cases

**Figure 2:**
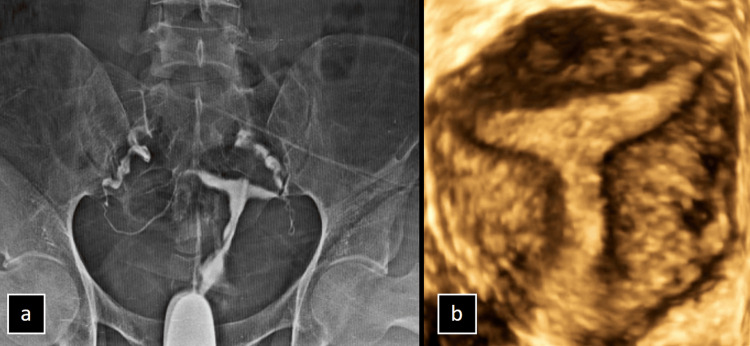
(a and b) Imaging of the T-Shaped Uterus in Patients With Infertility (a) Hysterosalpingography (HSG) image of a 35-year-old female with infertility reveals a small endometrial cavity with a T-shaped configuration, irregular and truncated bilateral fallopian tubes, and bilateral peritoneal spillage of contrast, suggestive of a "T-shaped" uterus. (b) 3D ultrasound image of a 31-year-old female revealing a “T-shaped" uterus.

The imaging findings revealed significant details of patients with infertility. Hysterosalpingography (HSG) of a 35-year-old female showed a small endometrial cavity with a T-shaped configuration and irregular, truncated bilateral fallopian tubes. The bilateral peritoneal spillage of contrast indicated a "T-shaped" uterus, a congenital anomaly frequently associated with in utero exposure to diethylstilbestrol (DES). Additionally, a 3D ultrasound of a 31-year-old female further confirmed the presence of the characteristic "T-shaped" uterus as shown in Figure 2.

**Figure 3:**
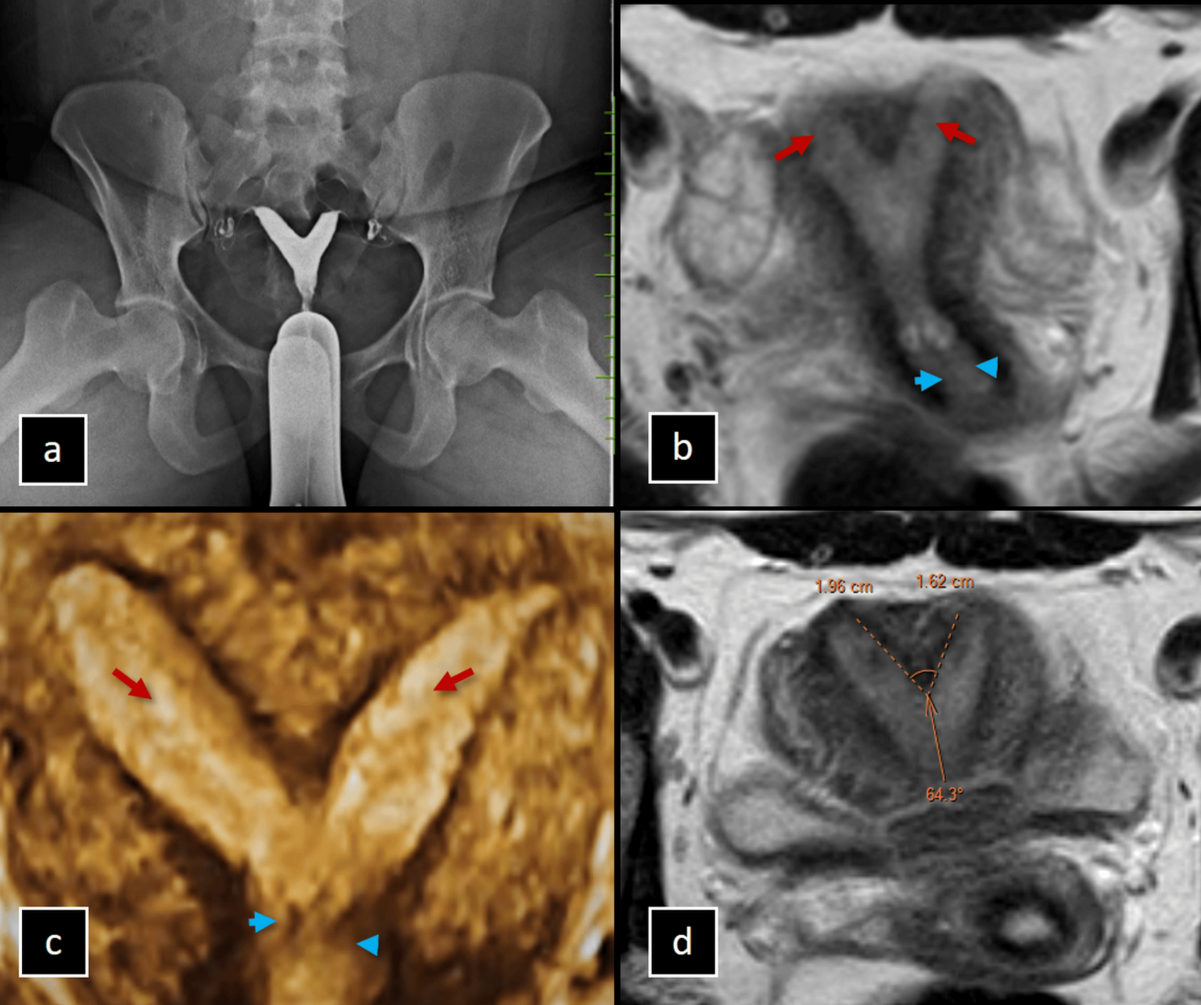
(a-d) Imaging of the Partial Septate Uterus in a Patient With Recurrent Pregnancy Loss. (a) Hysterosalpingogram (HSG) image of a 26-year-old female with recurrent pregnancy loss shows two endometrial cavities at the fundus level, joining above the internal os to form a single cervical canal, suggestive of a septate or bicornuate uterus. (b) MRI T2 oblique axial image demonstrates the two endometrial cavities (red arrows) joining above the internal os to form a single cervical canal (blue arrowheads). (c) 3D ultrasonogram demonstrates the two endometrial cavities (red arrows) joining above the internal os to form a single cervical canal (blue arrowheads). The external uterine contour appears mildly concave with an angle of 64 degrees between the horns. (d) MRI T2 oblique axial image demonstrates partial septate uterus.

The imaging findings in a 26-year-old female with recurrent pregnancy loss revealed two endometrial cavities at the fundus level that converge above the internal os to form a single cervical canal, suggestive of either a septate or bicornuate uterus. MRI further confirmed these results, showing the two endometrial cavities merging above the internal os into a single cervical canal. The external uterine contour appears mildly concave with an angle of 64 degrees between the horns, indicating a partial septate uterus as shown in Figure 3. 

**Figure 4:**
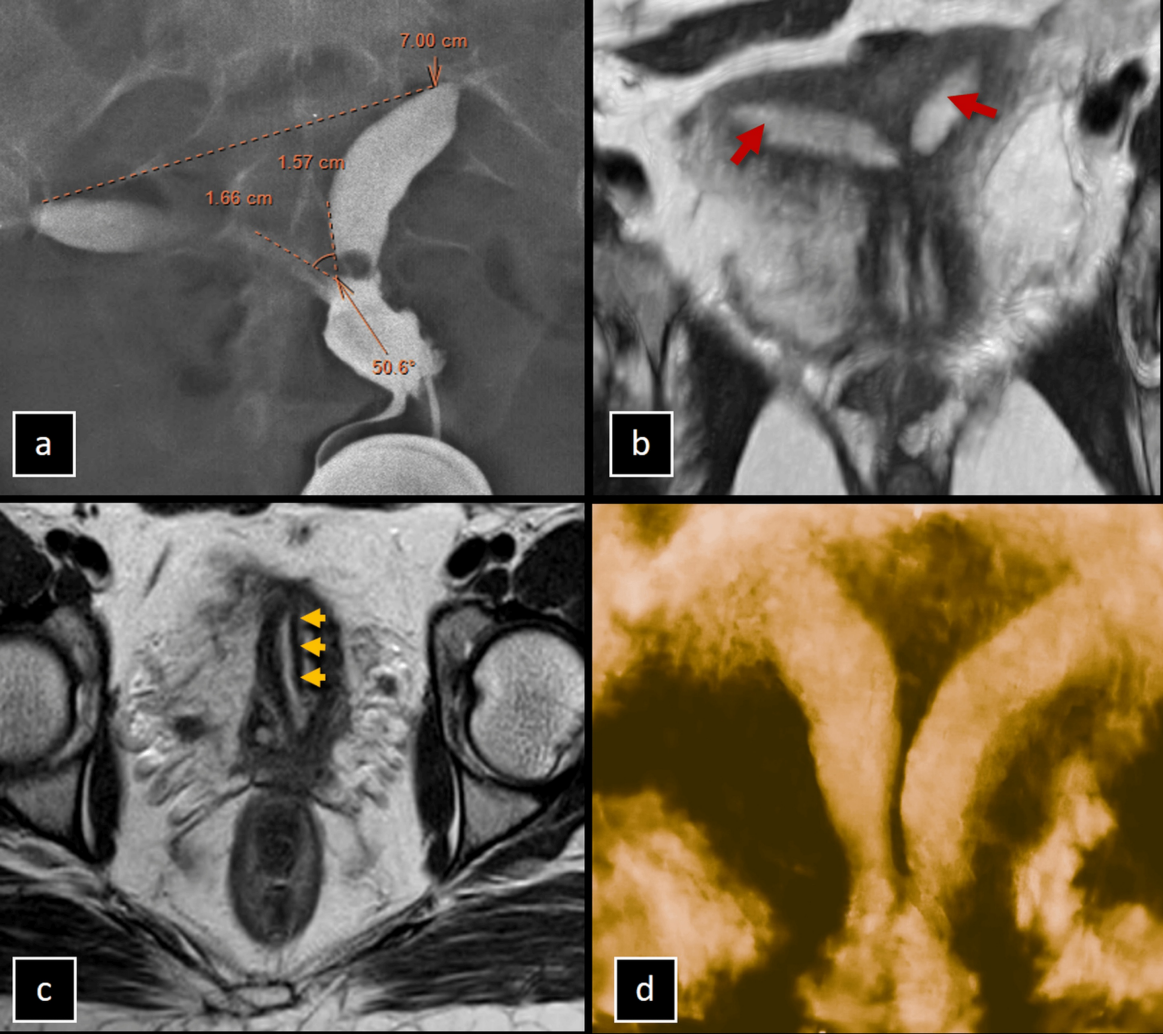
(a-d) Imaging of the Complete Septate Uterus in a Patient with Infertility. (a) Hysterosalpingogram (HSG) image of a 27-year-old female with infertility shows contrast opacification of a single cervical canal with two discrete, spaced uterine cavities, an inter-cornual distance of approximately 7 cm, and an inter-cornual angle of approximately 51 degrees, which is equivocal between bicornuate unicollis and complete septate uterus. (b) MRI oblique axial T2-weighted maximum intensity projection (MIP) and T2-weighted axial (c) images confirm the findings of two uterine cavities in the fundus (red arrows) with an intervening septum extending from the fundus to the cervix/vagina (yellow arrowheads). These features favor a diagnosis of a complete septate uterus. (d) 3D ultrasound provided additional information on the septum's thickness and vascularity.

The imaging findings of a 27-year-old female with infertility reveal contrast opacification of a single cervical canal with two discrete, spaced uterine cavities. The inter-cornual distance measures approximately 7 cm, and the inter-cornual angle is around 51 degrees, presenting an equivocal diagnosis between a bicornuate unicollis and a complete septate uterus. Further MRI evaluations, including oblique axial T2-weighted maximum intensity projection and T2-weighted axial images, confirm the presence of two uterine cavities in the fundus with an intervening septum extending from the fundus to the cervix and vagina. These features are indicative of a complete septate uterus. Additionally, 3D ultrasound provided further insights into the septum's thickness and vascularity as shown in Figure 4.

**Figure 5:**
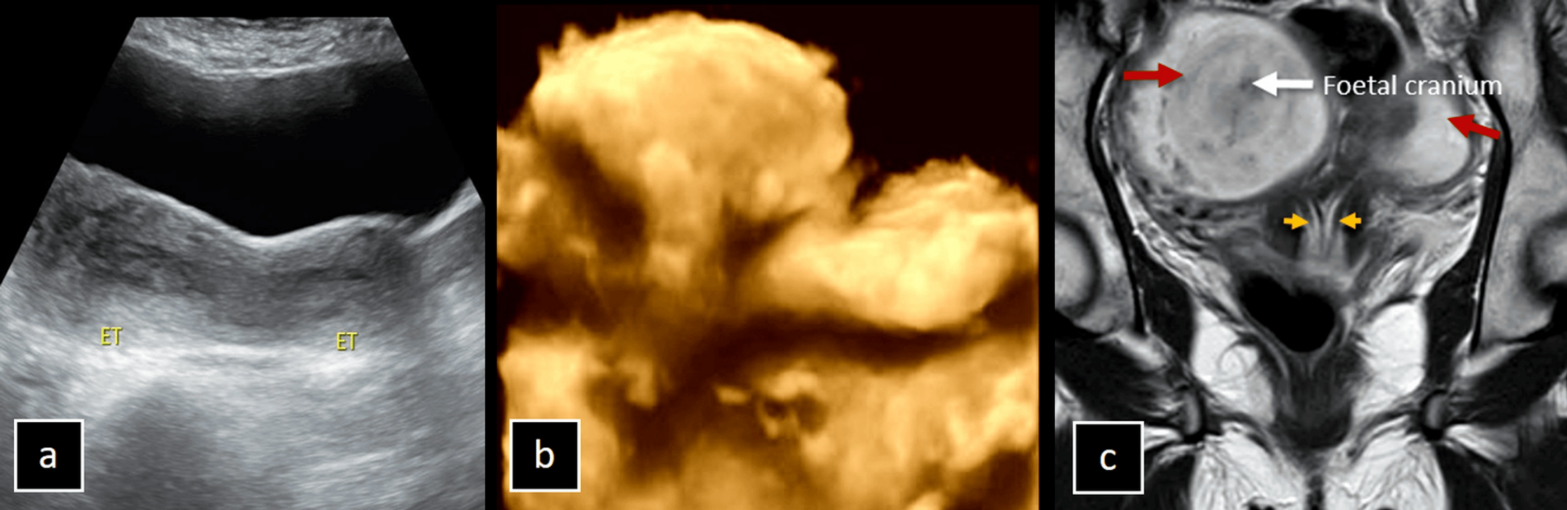
(a-c) Imaging of the Bicornuate Bicollis Uterus in a Patient With Abdominal Pain. Greyscale ultrasound (a) and 3D ultrasound (b) of a 19-year-old female during a routine abdominal ultrasound for evaluation of pain revealed an incidental finding of a bicornuate uterus with two separate cervices. Further MRI, T2 coronal section (c) during the antenatal period, demonstrates two divergent uterine horns (red arrows) with a solitary vagina but separate cervices (yellow arrowheads), consistent with a bicornuate bicollis uterus. A single intrauterine gestation is present in the right horn.

A 19-year-old female, evaluated for abdominal pain, was found to have an incidental bicornuate uterus with two separate cervices. Initial greyscale and 3D ultrasound examinations revealed this anomaly. Further MRI during the antenatal period confirmed the diagnosis, showing two divergent uterine horns with a solitary vagina but separate cervices, consistent with a bicornuate bicollis uterus. Additionally, a single intrauterine gestation was noted in the right horn as shown in Figure 5.

**Figure 6:**
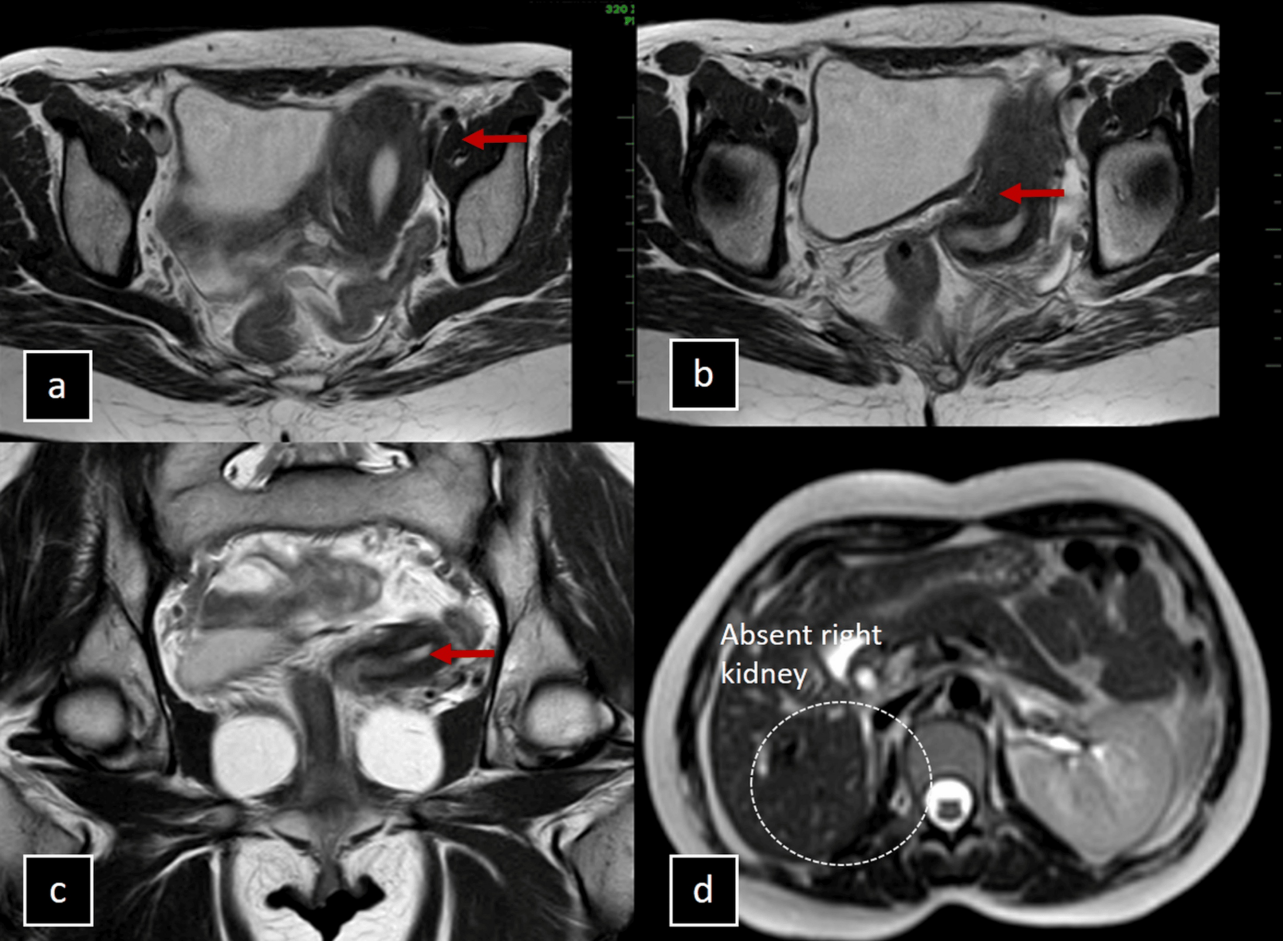
(a-d) Imaging of the True Unicornuate Uterus with No Rudimentary Horn in a 29-Year-Old Female. MRI images of a 29-year-old female. T2 axial sections (a, b), coronal section (c), and T2 axial section at the level of the bilateral kidneys show a uterus displaced to the left adnexa with no evidence of a rudimentary horn (red arrows) and an absent right ovary. Additionally, there is an absence of the right kidney (d). These findings are consistent with a Type IId True Unicornuate (Left) uterus with no rudimentary horn.

MRI imaging of a 29-year-old female revealed a uterus displaced to the left adnexa, with no evidence of a rudimentary horn and an absent right ovary. The T2 axial and coronal sections also showed an absence of the right kidney. These findings are consistent with a Type IId True Unicornuate (Left) uterus, characterized by the absence of a rudimentary horn and right kidney as shown in Figure 6.

**Figure 7:**
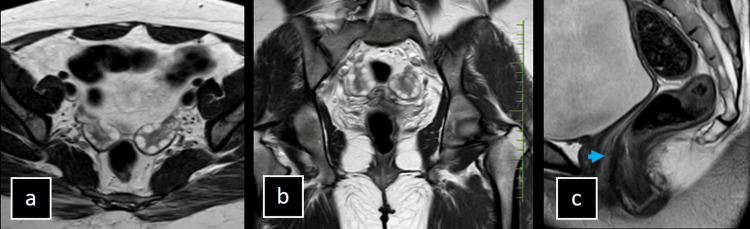
(a-c) Imaging of Mayer-Rokitansky-Küster-Hauser (MRKH) Syndrome in a Patient With Primary Amenorrhea. MRI of a 17-year-old female presenting with primary amenorrhea reveals non-visualization of the uterus, cervix, and upper two-thirds of the vagina, with a hypoplastic lower segment of the vagina. The bilateral ovaries appear normal. These findings are indicative of MRKH syndrome.

MRI of a 17-year-old female presenting with primary amenorrhea revealed non-visualization of the uterus, cervix, and upper two-thirds of the vagina, with a hypoplastic lower segment of the vagina. The bilateral ovaries appeared normal. These findings are indicative of Mayer-Rokitansky-Küster-Hauser (MRKH) syndrome as shown in Figure 7.

## Discussion

The accurate diagnosis and classification of Müllerian anomalies are crucial for appropriate management and improved reproductive outcomes. This study compared the diagnostic efficacy of 3D-US and MRI in detecting and classifying these anomalies, revealing important insights into the strengths and limitations of each modality. Our findings demonstrate the superior diagnostic accuracy of MRI compared to 3D-US in the evaluation of Müllerian anomalies, consistent with previous studies [25]. The higher sensitivity and specificity of MRI can be attributed to its excellent soft-tissue contrast and multiplanar imaging capabilities, which are particularly valuable in delineating complex uterine malformations [26]. MRI's ability to provide a comprehensive assessment of the internal and external uterine contour makes it the gold standard for differentiating between septate and bicornuate uteri, a distinction crucial for management decisions [27].

However, the diagnostic performance of 3D-US was also noteworthy, with good sensitivity and specificity. This aligns with recent advancements in 3D-US technology, which have significantly enhanced its role in gynecological imaging [10]. The real-time nature of 3D-US, combined with its ability to assess uterine vascularity, offers unique advantages, particularly in evaluating septal characteristics and planning surgical interventions [11]. The complementary nature of these modalities was evident in our study. While MRI excelled in providing a comprehensive overview of pelvic anatomy and associated anomalies, 3D-US offered valuable insights into functional aspects such as endometrial thickness and vascularity. This synergy suggests that a combined approach, utilizing both MRI and 3D-US, may provide the most comprehensive evaluation of Müllerian anomalies [12].

The application of both ESHRE/ESGE and ASRM classification systems in our study highlighted the complexities in categorizing Müllerian anomalies. The high inter-rater reliability (Cohen's kappa 0.85) indicates the robustness of these classification systems when applied by experienced radiologists. However, the existence of unclassified cases (7.3%) underscores the need for ongoing refinement of classification criteria to encompass the full spectrum of anatomical variations [16,17]. The challenges in differentiating between certain anomaly types, particularly septate and bicorporeal uteri, were evident in our study. MRI's superior ability to visualize the external fundal contour proved crucial in these cases, supporting its role as the definitive imaging modality for complex anomalies [13]. Conversely, 3D-US demonstrated particular strength in evaluating septal thickness and vascularity, information critical for surgical planning in cases of septate uteri [14].

Clinical correlations and management implications

The significant associations found between specific anomaly types and presenting symptoms provide valuable insights for clinical practice. The higher prevalence of primary amenorrhea in aplastic and hemi-uteri and the strong correlation between septate uteri and recurrent pregnancy loss align with established literature [3,4]. These correlations emphasize the importance of accurate imaging in guiding management strategies and counseling patients about reproductive outcomes.

The role of imaging extends beyond diagnosis to treatment planning. For instance, the detailed assessment of septal vascularity by 3D-US can inform surgical approaches in metroplasty procedures. Similarly, MRI's ability to detect associated renal anomalies is crucial for comprehensive patient management [5].

Technological advancements and future directions

The evolving landscape of imaging technology presents exciting possibilities for enhancing the diagnosis of Müllerian anomalies. Advanced MRI techniques, such as diffusion-weighted imaging and dynamic contrast-enhanced sequences, offer potential for further improving tissue characterization and functional assessment [18]. Similarly, the integration of artificial intelligence and machine learning algorithms with 3D-US may enhance its diagnostic accuracy and reduce operator dependence [19]. The development of fusion imaging techniques, combining real-time 3D-US with previously acquired MRI data, represents a promising avenue for leveraging the strengths of both modalities. This approach could provide a comprehensive, real-time assessment of uterine morphology and function, potentially streamlining the diagnostic process and improving surgical planning [20].

Several limitations of our study warrant consideration. The retrospective design may have introduced selection bias, and the single-center nature of the study may limit generalizability. Future prospective, multi-center studies could address these limitations and provide more robust evidence. Additionally, while our study focused on imaging accuracy, future research should explore the impact of different imaging strategies on clinical outcomes, including successful pregnancy rates and surgical outcomes. Long-term follow-up studies comparing management decisions based on MRI versus 3D-US findings could provide valuable insights into the clinical implications of these imaging modalities. The cost-effectiveness of different imaging approaches in the diagnosis of Müllerian anomalies is another area deserving further investigation. While our preliminary analysis showed 3D-US to be more cost-effective, a comprehensive economic evaluation considering long-term clinical outcomes is needed to inform healthcare policy and resource allocation.

While MRI remains the gold standard, especially for complex cases, 3D-US offers a valuable first-line imaging option, particularly in settings where MRI access is limited. A stepwise approach, starting with 3D-US and progressing to MRI for complex or equivocal cases, may optimize resource utilization while maintaining diagnostic accuracy. The complementary nature of MRI and 3D-US suggests that, when feasible, a combined approach may provide the most comprehensive evaluation. This is particularly relevant in cases where surgical intervention is being considered, as it allows for detailed pre-operative planning. The high inter-rater reliability achieved in our study underscores the importance of standardized classification systems. Clinicians and radiologists should be well-versed in both ESHRE/ESGE and ASRM classification systems to ensure consistent reporting and facilitate effective communication across healthcare teams. The strong correlations between specific anomaly types and presenting symptoms highlight the importance of considering clinical presentation when selecting and interpreting imaging studies. This symptom-guided approach can enhance diagnostic efficiency and accuracy. Given the complex nature of Müllerian anomalies and their impact on reproductive health, a multidisciplinary approach involving radiologists, gynecologists, and reproductive endocrinologists is crucial for optimal patient management.

Challenges in imaging interpretation

Our study also highlighted several challenges in the imaging interpretation of Müllerian anomalies; one of them was distinguishing between minor septal indentations and true uterine anomalies which can be challenging, particularly on 3D-US. This underscores the need for experienced operators and the potential benefit of MRI in equivocal cases. The presence of uterine fibroids or endometriosis can complicate image interpretation, particularly for 3D-US. In such cases, MRI's superior soft-tissue contrast becomes particularly valuable. The uterus's changing appearance throughout the menstrual cycle can affect imaging interpretation. Standardizing the timing of imaging studies relative to the menstrual cycle may improve consistency in diagnosis. Uncommon variants of Müllerian anomalies may not fit neatly into existing classification systems, presenting diagnostic challenges. Continuous education and case-based learning are essential for radiologists to stay updated on the full spectrum of anomalies.

Emerging technologies and future perspectives looking ahead 

Several emerging technologies and research directions hold promise for further advancing the diagnosis and management of Müllerian anomalies. These include advanced MRI Techniques like Functional MRI techniques, such as diffusion tensor imaging, may provide insights into myometrial fiber orientation, potentially aiding in the differentiation between septate and bicornuate uteri [21], Contrast-Enhanced 3D-US with the development of contrast agents for 3D-US could enhance vascular mapping, improving the assessment of septal vascularity and aiding in surgical planning [22], artificial intelligence (AI) integration which includes machine learning algorithms could potentially enhance the accuracy of both MRI and 3D-US interpretation, assisting in anomaly classification and reducing inter-observer variability [23], virtual and augmented reality which could provide immersive 3D visualizations of complex anomalies, enhancing surgical planning and patient education [24], and molecular imaging for the assessment of endometrial receptivity in women with Müllerian anomalies, providing valuable information for fertility management [25].

Global perspectives and healthcare disparities

It is important to consider the global context of Müllerian anomaly diagnosis and management. While our study was conducted in a tertiary care center with access to advanced imaging modalities, many healthcare settings worldwide may not have such resources. The role of 3D-US as a more accessible and cost-effective option becomes particularly significant in resource-limited environments. Future research should explore strategies for optimizing the diagnostic accuracy of 3D-US in these settings, potentially through telemedicine consultations or AI-assisted interpretation [26].

## Conclusions

This study demonstrates the complementary roles of 3D-US and MRI in the diagnosis and classification of Müllerian anomalies. While MRI showed superior overall diagnostic accuracy, particularly for complex anomalies, 3D-US proved to be a valuable tool, especially for assessing septal characteristics and uterine vascularity. The integration of these imaging modalities into clinical practice enhances diagnostic precision and facilitates tailored management strategies. The strong associations observed between specific anomaly types and clinical presentations underscore the importance of accurate imaging in guiding patient management and counseling. The high inter-rater reliability achieved using standardized classification systems highlights the value of consistent reporting methodologies.

Looking ahead, emerging technologies and advanced imaging techniques promise to further refine our ability to diagnose and manage Müllerian anomalies. However, it is crucial to consider global healthcare disparities and work toward developing accessible and accurate diagnostic approaches for all healthcare settings. In conclusion, a comprehensive, patient-centered approach that leverages the strengths of both 3D-US and MRI, guided by clinical presentation and standardized classification systems, offers the best path forward in optimizing care for women with Müllerian anomalies. Continued research, technological innovation, and global collaboration will be key to advancing our understanding and management of these complex reproductive tract disorders.
